# Dietary Fiber and Its Source Are Associated with Cardiovascular Risk Factors in Korean Adults

**DOI:** 10.3390/nu13010160

**Published:** 2021-01-06

**Authors:** SuJin Song, YoonJu Song

**Affiliations:** 1 Department of Food and Nutrition, Hannam University, Daejeon 34054, Korea; sjsong@hnu.kr; 2 Department of Food Science and Nutrition, The Catholic University of Korea, Gyeonggi 14662, Korea

**Keywords:** total dietary fiber, fruit fiber, obesity, metabolic syndrome, hypertension, Korean adults

## Abstract

We examined the associations of dietary fiber and its source with cardiovascular risk factors in Korean adults. This cross-sectional study involved 16,792 adults from the 2013–2018 Korea National Health and Nutrition Examination Survey data. Dietary data were obtained using a 24 h recall method and used to evaluate intakes of total dietary fiber and its source and fruit consumption. Cardiovascular risk factors included obesity, abdominal obesity, metabolic syndrome, hypercholesterolemia, hypertension, and type 2 diabetes. Multiple logistic regression was used to examine the associations of dietary fiber and its source with cardiovascular risk factors by sex. Total fiber and fruit fiber intake in men were inversely associated with metabolic syndrome (Q5 vs. Q1: odds ratios (OR) = 0.69, 95% confidence intervals (CI) = 0.53–0.92 for total fiber; Q4 vs. Q1: OR = 0.76, 95% CI = 0.61–0.93 for fruit fiber). Among women, a higher intake of fruit fiber was related to a reduced prevalence of obesity (Q4 vs. Q1: OR = 0.85, *p* trend = 0.029) and abdominal obesity (Q4 vs. Q1: OR = 0.82, *p* trend = 0.026). Total fruit and whole fruit consumption was inversely associated with obesity, abdominal obesity, and metabolic syndrome in men and hypertension in women. The amount and sources of fiber are associated with metabolic diseases in Korean adults and should be considered in the context of overall dietary quality.

## 1. Introduction

Dietary fiber is derived from dietary carbohydrate, which is an important source of energy for humans. The findings regarding the relationship between total carbohydrate intake and the risk for cardiovascular diseases (CVD) have been inconsistent, as dietary carbohydrate is present in various foods and has different glycemic effects [1]. In terms of food source in carbohydrates, whole grains and fruits have been reportedly associated with a reduced risk for CVD [2,3]; by contrast, refined grains and sugar-sweetened beverages have been reported to show an increased risk for metabolic diseases [4,5]. Thus, carbohydrate quality is a focus of research related to disease prevention and management [1].

In a recent comprehensive meta-analysis of carbohydrate quality measures, dietary fiber, whole grains, and the dietary glycemic index were reviewed [6]. While the dietary glycemic index was slightly or not associated with the risk for noncommunicable diseases, high fiber consumption or habitual daily consumption of whole grains was associated with a considerable reduction in the risk for noncommunicable diseases [6]. Another meta-analysis of 10 cohort studies also reported that whole grain consumption corresponds with a reduced risk of cardiovascular disease, cancer, and mortality [7]. However, there is a limitation of no standard definition for whole grains [8].

Dietary fiber has recently been a focus of research attention due to the wide availability of refined grains and the importance of the human gut microbiome [9]. A study of human subjects showed that dietary fibers from beans, fruits, and vegetables were associated with the gut microbiome composition [9]. These microbiotal changes have been reported to be associated with obesity and related disorders, such as metabolic syndrome [10].

Regarding high fiber intake and health outcomes, a high fiber intake is reportedly associated with desirable effects on cardiovascular risk factors, such as blood pressure and blood lipids [11,12,13]. A meta-analysis identified an inverse association between dietary fiber intake and metabolic syndrome in cross-sectional studies but not in cohort studies due to insufficient data [14].

Because dietary fiber is a component of dietary carbohydrate, its sources as well as its proportion of total carbohydrate intake are also important for evaluating dietary fiber. The American Heart Association recommends the consumption of fiber-rich whole grains and at least 1.1 g of fiber per 10 g of carbohydrate [15]. Fontanelli et al. [16] reported that grain consumption with at least 1 g of fiber per 10 g of carbohydrate (a 10:1 ratio) was associated with higher nutritional quality and inversely associated with cardiometabolic risk factors. AlEssa et al. [17] reported that the risk for coronary heart disease among men and women in the US was associated with the carbohydrate-to-cereal fiber ratio but not the carbohydrate-to-total fiber ratio in fully adjusted models. A previous prospective study on sources of dietary fiber reported that fruit fiber protects against metabolic syndrome, but other sources of fiber do not [18]. In a cohort study of Japanese adults, cereal and fruit fiber but not vegetable fiber intake was associated with a reduced risk for CVD mortality [19].

Previous work in Korean adults focused on the relationships between carbohydrate quantity (i.e., total carbohydrate intake, percentage of energy from carbohydrate, dietary glycemic index and glycemic load, and refined grains consumption) and metabolic syndrome and found that the percentage of energy from carbohydrate and refined grains consumption was positively associated with the prevalence of metabolic syndrome [20]. However, few epidemiologic studies have evaluated the associations of dietary fiber and its source with metabolic diseases, such as obesity, hypertension, and type 2 diabetes, in the Korean adult population, whose diets typically comprise a high carbohydrate content due to carbohydrate-rich staple foods. Therefore, we investigated the associations between dietary fiber and its source with cardiovascular risk factors in Korean adults based on national health and nutrition data.

## 2. Materials and Methods

### 2.1. Data and Subjects

This study was based on data from the 2013–2018 Korea National Health and Nutrition Examination Survey (KNHANES). The KNHANES is a cross-sectional survey, which is conducted by the Korea Centers for Disease Control and Prevention with the aim of regularly monitoring the nutritional health status of Koreans. This survey includes nationally representative samples based on a stratified, multistage probability sampling design. Health and nutrition related information are collected through health interviews, health examinations, and nutrition surveys. A detailed explanation is available elsewhere [21].

**Figure 1:**
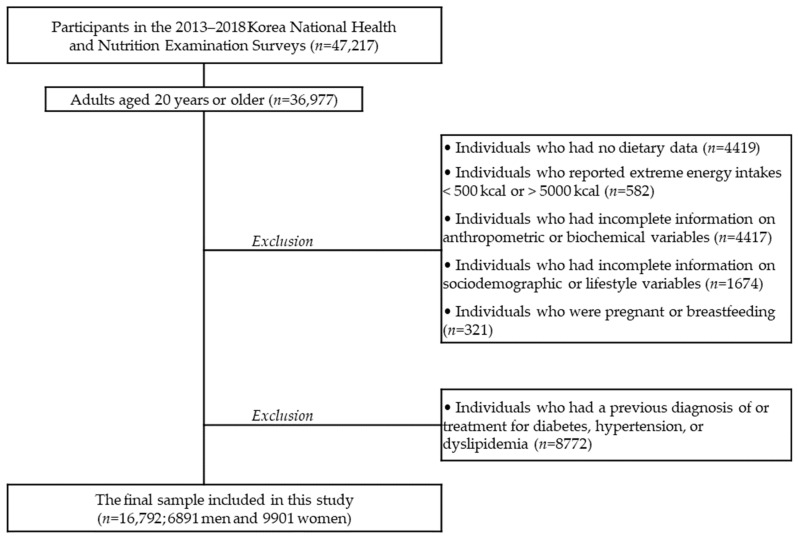
Selection of the study population.

A total of 36,977 adults aged ≥20 years from the 2013–2018 KNHANES were eligible for this study. Among the 36,977 adults, individuals who had no dietary data (*n* = 4419), reported extreme energy intakes (<500 kcal or >5000 kcal; *n* = 582), had incomplete information on anthropometric or biochemical measures (*n* = 4417), had incomplete information on sociodemographic or lifestyle variables (*n* = 1674), and/or were pregnant or breastfeeding (*n* = 321) were excluded from the analyses. In addition, individuals who had been diagnosed or treated for dyslipidemia, diabetes, or hypertension were excluded (*n* = 8772) because the diseases can alter individuals’ diet. The final sample included 16792 Korean adults (6891 men and 9901 women). Figure 1 shows the flow of the study population selection. The study was conducted in accordance with the Declaration of Helsinki and was approved by the Institutional Review Board of the Korea Centers for Disease Control and Prevention (IRB No. 2013-07CON-03-4C, 2013-12EXP-03-5C, 2018-01-03-P-A). Written informed consent was obtained from each subject.

### 2.2. Assessment of Dietary Fiber and Its Source

Dietary data were obtained using a single 24 h dietary recall method. During the nutrition survey, the 24 h dietary recall was administered by trained interviewers at the participants’ households. A standardized and structured interview was conducted for each participant. Participants were asked for detailed information on all foods and beverages consumed in the past 24 h, and food models, pictures, and other visual aids were used to help participants recall quantities of foods and beverages consumed. Dietary data obtained were linked to a food composition database, which was established by the Rural Development Administration of Korea [22,23], and then energy and nutrient intake from foods and beverages was determined for each participant. A food group code provided in the dietary data of KNHANES was used to calculate the intake of a fiber source, including cereal, vegetable, and fruit. This study evaluated intakes of total dietary fiber, carbohydrate-to-total fiber ratio, cereal fiber, vegetable fiber, and fruit fiber as well as fruit consumption. Each dietary measure was categorized into quintile groups (or quartile groups) by sex for further analyses.

### 2.3. Assessment of Cardiovascular Risk Factors

Anthropometric and biochemical variables were measured by trained medical personnel with standard methods during the health examination. Body mass index (BMI) was calculated from the measured height and weight (kg/m^2^). Waist circumference was measured to the nearest 0.1 cm using a measuring tape at the umbilical level in a standing position. Blood pressure was measured three times, and the average of the last two values was used. Venous blood samples were collected from subjects after they had fasted for at least 8 h. Levels of total cholesterol, triglycerides, high-density lipoprotein (HDL)-cholesterol, and fasting blood glucose were measured using enzymatic methods with a 7600–210 automatic analyzer (Hitachi, Tokyo, Japan) in a certified clinical laboratory.

Cardiovascular risk factors evaluated in this study were obesity, abdominal obesity, metabolic syndrome, hypercholesterolemia, hypertension, and type 2 diabetes. Obesity was defined as BMI ≥ 25 kg/m^2^ [24]. Abdominal obesity was determined based on a cutoff for Koreans as a waist circumference ≥90 cm in men and ≥85 cm in women [25]. Metabolic syndrome was determined by the National Cholesterol Education Program Adult Treatment Panel III criteria [26] if any three or more of the following components were present: abdominal obesity (waist circumference ≥90 cm in men and ≥85 cm in women); hypertriglycerides (triglycerides ≥150 mg/dL); low HDL-cholesterol (HDL-cholesterol <40 mg/dL in men and <50 mg/dL in women); elevated blood pressure (systolic blood pressure ≥130 mmHg or diastolic blood pressure ≥85 mmHg); and elevated blood glucose (fasting blood glucose ≥100 mg/dL). Hypercholesterolemia was defined as total cholesterol ≥240 mg/dL [26]. The definition of hypertension was from the Korean Hypertension Association [27]: systolic blood pressure ≥140 mmHg or diastolic blood pressure ≥90 mmHg. Type 2 diabetes was determined as fasting blood glucose ≥126 mg/dL [28].

### 2.4. Measurements of Sociodemographic and Lifestyle Variables

Sociodemographic (e.g., age, living area, education, and household income) and lifestyle (e.g., smoking, alcohol drinking, and physical activity) information was collected in the health interview part. Current smokers were individuals who had smoked ≥100 cigarettes during their lifetime and were smoking regularly or occasionally at the time of the survey. Current alcohol drinkers were individuals who drank ≥1 glass of alcohol per month in the previous year. Physical activity was determined as “yes” if a subject engaged in walking exercise for ≥30 min on at least 5 days during the previous week.

### 2.5. Statistical Analyses

Statistical analyses were conducted using Statistical Analysis Systems (SAS) software, version 9.4 (SAS Institute, Cary, NC, USA). All the analyses performed in this study accounted for the effects of the complex sampling design and used appropriate sampling weights. Characteristics of the study population were presented as means with standard errors (SE) for continuous variables and frequencies and percentages for categorical variables. The *t*-test and Rao–Scott chi-square test were used to analyze the differences in these variables by sex. The distribution of dietary fiber and its source by quintile (or quartile) of each dietary measure was expressed as the median and interquartile range. Multiple logistic regression analyses were performed to estimate odds ratios (ORs), 95% confidence intervals (CIs), and *P* trend values for cardiovascular risk factors by quintile (or quartile) of each dietary measure, taking the lowest quintile (or quartile) group as the reference group. To calculate the *P* trend values across quintiles (or quartiles) of dietary measures, the median intake of a dietary measure in each quintile (or quartile) was entered as a continuous variable in the logistic regression model. In all of the models, age (continuous), living area (urban or rural), education (elementary school, middle school, high school, or college or more), household income (lowest, medium-low, medium-high, or highest), current smoking (yes or no), current alcohol drinking (yes or no), physical activity (yes or no), BMI (continuous), and total energy intake (continuous) were controlled as potential confounding variables. Values of *p* < 0.05 were considered indicative of statistical significance.

## 3. Results

### 3.1. Characteristics of the Subjects by Sex

Table 1 shows the characteristics of the study subjects by sex. The mean ages of men and women were 41.4 and 42.2 years, respectively (*p* < 0.001). In men, the proportions of individuals who live in a rural area and have a higher education level were higher compared to those in women. The proportions of current smokers, current alcohol drinkers, and individuals who engage in physical activity were higher in men than in women. Energy and nutrient intakes showed significant differences between men and women. Men showed higher intakes of total fiber, cereal fiber, and vegetable fiber and a higher carbohydrate-to-total fiber ratio, but a lower intake of fruit fiber, than women.

### 3.2. Distribution of Dietary Fiber and Its Source by Sex

Table 2 lists the intakes of dietary fiber and its source by sex. Each dietary measure was grouped into quintiles or quartiles by sex, and the median and range for each quintile (quartile) were calculated. The intake of total fiber, cereal fiber, and vegetable fiber and carbohydrate-to-total fiber ratio showed higher median values of quintile groups in men than in women. Compared to men, women had a higher median intake of fruit fiber in the quintile (quartile) groups.

### 3.3. Association of Dietary Fiber and Its Source with Cardiovascular Risk Factors in Men

The associations of dietary fiber and its source with cardiovascular risk factors in men are shown in Table 3. In men, total dietary fiber and fruit fiber intake was inversely associated with the prevalence of metabolic syndrome. In the highest quintile (quartile), the OR for metabolic syndrome was 0.69 (95% CI = 0.53–0.92, *p* trend = 0.004) and 0.76 (95% CI = 0.61–0.93, *p* trend = 0.011) for total fiber and fruit fiber intake, respectively. A higher ratio of carbohydrate to total fiber showed an inverse relationship with obesity and abdominal obesity. Vegetable fiber intake was positively associated with obesity. Fruit fiber intake was inversely associated with the prevalence of abdominal obesity (Q4 vs. Q1: OR = 0.74, 95% CI = 0.62–0.88, *p* trend = 0.001) and hypertension (Q4 vs. Q1: OR = 0.77, 95% CI = 0.61–0.97, *p* trend = 0.035) but positively associated with hypercholesterolemia.

### 3.4. Association of Dietary Fiber and Its Source with Cardiovascular Risk Factors in Women

Table 4 presents the associations between dietary fiber and its source and cardiovascular risk factors in women. In women, a high intake of fruit fiber was associated with a reduced prevalence of obesity (Q4 vs. Q1: OR = 0.85, 95% CI = 0.73–1.00, *p* trend = 0.029) and abdominal obesity (Q4 vs. Q1: OR = 0.82, 95% CI = 0.67–0.99, *p* trend = 0.026). In the highest quartile of fruit fiber intake of women, the OR for hypertension was 0.70 (95% CI = 0.54–0.90) and the OR for type 2 diabetes was 0.51 (95% CI = 0.28–0.91). Other dietary measures were not associated with cardiovascular risk factors in women.

### 3.5. Association of Fruit Consumption with Cardiovascular Risk Factors in Men and Women

Table 5 shows the associations between fruit consumption and cardiovascular risk factors by sex. Among men, total fruit and whole fruit consumption was inversely associated with obesity, abdominal obesity, and metabolic syndrome. A higher intake of total fruit consumption was related to the reduced prevalence of hypertension in both sexes (Q4 vs. Q1: OR = 0.78, 95% CI = 0.62–0.98 for men; OR = 0.68, 95% CI = 0.53–0.88 for women). Whole fruit intake was inversely associated with hypertension in women.

## 4. Discussion

This large-sample cross-sectional study of Korean adults examined the associations of dietary fiber and its source with cardiovascular risk factors, including obesity and metabolic syndrome. Total dietary fiber and fruit fiber intake were inversely associated with metabolic syndrome in men, and fruit fiber intake was inversely associated with abdominal obesity and hypertension in both men and women.

Men in the highest quintile of total fiber intake were 31% less likely to have metabolic syndrome than those in the lowest quintile. Our results are in line with a meta-analysis of dietary fiber and metabolic syndrome, which reported a pooled OR of 0.70 (95% CI = 0.61–0.82) for cross-sectional studies [14]. In addition, total fiber is reportedly inversely associated with the risk for coronary heart disease [17], insulin resistance [29,30], high blood pressure [12], weight gain [31,32], and mortality from CVD [33]. The mechanism of the benefit of dietary fiber for metabolic diseases may be high satiety, a reduced nutrient absorption rate, energy dilution, or change in the gut microbiota [34]. However, the effect of dietary fiber on cardiometabolic risk factors is also reported to differ according to its physicochemical properties, amount, and source.

Given that the recommended intake of dietary fiber for Koreans is 25 g in men and 20 g in women [35], the average intake of 26.0 g in men and 22.6 g in women in this study was adequate. Indeed, it was comparable or slightly higher than that in Japanese or Chinese adults, whose diet is similar to Koreans. Nakaji et al. [36] reported a fiber intake of 15–20 g/d for Japanese adults in the 1980s and 1990s, and Wang et al. [37] reported a fiber intake of 17–19 g/d for Chinese adults aged ≥45 years from 1991 to 2011. However, in this study, the average carbohydrate-to-total fiber ratio was 15.4 in men and 14.2 in women. This value is lower than that of Japanese adults (15.6) [29] but higher than that of US adults (11.3) [17].

The American Heart Association recommends an overall healthy dietary pattern to prevent and manage CVD, including at least 1.1 g fiber per 10 g of carbohydrate [15]. Fontanelli et al. [16] reported that foods meeting the <10:1 ratio showed high nutritional quality, and higher intakes of these foods are associated with cardiometabolic risk factors. Regarding sources of cereal fiber, the staple food for Korean adults is white rice, which means high consumption of grains but low intake of cereal fiber. Therefore, in Korean adults, dietary fiber intake should be evaluated according to carbohydrate quality, including the sources of dietary fiber.

The relationship between dietary fiber sources and metabolic diseases was inconsistent. A study of US adults reported that cereal fiber intake was inversely associated with a lower risk for coronary heart disease, whereas the ratio of carbohydrate-to-cereal fiber was significantly associated with an increased risk for coronary heart disease [17]. However, total dietary fiber showed no such association [17]. Another study of US adults in the Framingham Offspring cohort showed that the highest quintile of cereal fiber intake was associated with a 68% reduction in the prevalence of metabolic syndrome but the intakes of total fiber, vegetable fiber, fruit fiber, and legume fiber were not [30]. A study of European adults showed that total fiber intake was inversely associated with subsequent weight and waist circumference changes over 6.5 years, but fruit or vegetable fiber intake showed no association with weight change [31].

In terms of fiber source, we found that fruit fiber intake showed a significant inverse association with cardiovascular risk factors, including obesity, hypertension, and type 2 diabetes, in Korean adults. Fruit fiber intake has been reported to be significantly and inversely associated with metabolic syndrome in Iranian adults [18,38], with insulin resistance in US adults [30], and with mortality from CVD in Japanese adults [19]. In the Tehran Lipid and Glucose Study, the intakes of total fiber, fruit fiber, cereal, and legume fiber were significantly associated with a reduced prevalence of metabolic syndrome. However, after three years of follow-up, only fruit fiber intake protected against metabolic syndrome [18,38].

Other than fruit fiber, fruit consumption has been reported to have a beneficial effect on metabolic diseases in Korean adults, with studies reporting that a fruit-and-dairy dietary pattern is inversely associated with metabolic syndrome [39], higher fruit consumption is associated with a lower risk for incident hypertension [40], and whole fruit consumption is associated with a reduced prevalence of obesity [41]. Considering fruit consumption was mainly composed of whole fruit consumption in this study population, whole fruit is a key component of dietary fiber intake in Koreans, and the health benefit of fruit is attributable to fruit fiber. As dietary fiber from beans, fruits, and vegetables has been reported to relate to the gut microbiome composition [9], further studies to elucidate the effect of dietary fiber on metabolic diseases through the gut microbiome will be necessary. An intervention study for Korean adults showed that the gut microbiome and microbial metabolites were significantly different between the typical Korean diet and the typical American diet in a four-week crossover clinical trial [42], in which dietary fiber intakes in two diets were distinct (29 g vs. 24 g) [43]. In our study, fruit fiber intake (coefficient of variation (CV) = 170) showed greater variation compared to cereal fiber (CV = 93.1) or vegetable fiber (CV = 75.1) intake. According to Chen et al., the strength of the association increases as the difference between the highest and lowest category of dietary fiber increases [14].

Substantial evidence shows that dietary patterns high in dietary fiber and its major sources, such as whole grains, vegetables, and fruits, are associated with reduced risks for CVD. A recent review reported consistent beneficial effects of a Mediterranean diet and a Dietary Approaches to Stop Hypertension (DASH) diet on cardiovascular risk factors [44]. Particularly, diets high in dietary fiber are also rich in phytochemicals, including carotenoids, flavonoids, and isoflavone. The positive effects of dietary fiber on CVD risk factors observed in this study could be related to these phytochemicals [45,46,47]. A high intake of isoflavone and tofu was inversely associated with the incidence of coronary heart disease in the US cohort studies [47].

This study had several limitations. First, because of the cross-sectional design, we could not evaluate the causality of the relationship between dietary variables and cardiovascular risk factors. A longitudinal study is needed to confirm that association. Second, one-day dietary recall did not represent the usual intake of the study population. However, the quintile (or quartile) groups were used to examine the association with cardiovascular risk factors. Third, insoluble/soluble fiber intake could not be determined because of the lack of data. Instead, food sources were examined to identify the effects of the types of dietary fiber on cardiovascular risk factors. Nevertheless, the strengths of the study were the use of a large and nationally representative sample and that this was the first exploration of the associations of dietary fiber and its source with cardiovascular risk factors among Korean adults.

## 5. Conclusions

Among carbohydrate quality measures, our results suggest that higher intake of dietary fiber, particularly fruit fiber, ameliorates cardiovascular risk factors, such as obesity, metabolic syndrome, and hypertension, in Korean adults. In the context of overall carbohydrate intake, the amount and source of dietary fiber should be considered to improve dietary quality and reduce the risk of CVD. Further studies should evaluate the mechanism underlying the role of dietary fiber and its source in the development of CVD.

## Figures and Tables

**Table 1:** Characteristics of the study subjects by sex ^1^.

	Total (*n* = 16,792)		Men (*n* = 6891)		Women (*n* = 9901)		
	*n*	%	*n*	%	*n*	%	*p*-value ^2^
Age							
20–29 years	2644	23.1	1170	24.5	1474	21.6	<0.001
30–49 years	7882	48.4	3001	47.8	4881	48.9	
50–64 years	4254	21.8	1678	20.6	2576	23.0	
≥65 years	2012	6.8	1042	7.1	970	6.5	
Living area							
Urban	14,020	86.9	5664	86.1	8356	87.6	0.005
Rural	2772	13.1	1227	13.9	1545	12.4	
Education							
Elementary school or less	1987	7.6	708	6.0	1279	9.2	<0.001
Middle school	1353	6.6	554	6.1	799	7.2	
High school	5989	38.4	2481	39.6	3508	37.3	
College or more	7463	47.3	3148	48.4	4315	46.2	
Household income							
Lowest	1981	9.8	817	9.3	1164	10.2	0.098
Medium-low	4041	23.5	1655	23.1	2386	23.9	
Medium-high	5161	31.9	2145	32.6	3016	31.2	
Highest	5609	34.9	2274	35.0	3335	34.8	
Current smoking							
Yes	3077	22.5	2575	39.1	502	5.7	<0.001
No	13,715	77.5	4316	60.9	9399	94.3	
Current alcohol drinking							
Yes	9694	62.3	4967	73.7	4727	50.8	<0.001
No	7098	37.7	1924	26.3	5174	49.2	
Physical activity							
Yes	6565	40.8	2776	41.8	3789	39.7	0.024
No	10,227	59.2	4115	58.2	6112	60.3	
	Mean	SE	Mean	SE	Mean	SE	*p*-value
Energy and nutrient intakes							
Total energy intake (kcal)	2092	8.6	2411	12.8	1770	8.1	<0.001
% of energy from carbohydrate	63.2	0.1	62.4	0.2	63.9	0.1	<0.001
% of energy from fat	21.7	0.1	22.0	0.1	21.4	0.1	<0.001
% of energy from protein	15.1	0.1	15.6	0.1	14.7	0.1	<0.001
Total fiber (g)	24.3	0.1	26.0	0.2	22.6	0.2	<0.001
Carbohydrate: Total fiber	14.8	0.1	15.4	0.1	14.2	0.1	<0.001
Cereal fiber (g)	5.3	0.04	5.8	0.1	4.9	0.1	<0.001
Vegetable fiber (g)	7.8	0.1	8.7	0.1	6.8	0.1	<0.001
Fruit fiber (g)	3.4	0.1	2.9	0.1	3.8	0.1	<0.001

SE, standard error. ^1^ All of the analyses accounted for the effects of the complex sampling design and appropriate sampling weights of the national survey using the PROC SURVEY procedures in the SAS software. ^2^ *p*-values were calculated by the Rao–Scott chi-square test for categorical variables and by a *t*-test for continuous variables to test the differences in the variables by sex.

**Table 2:** Distribution of dietary fiber and its source by sex ^1^.

	Quintile (Quartile) of Dietary Fiber and Its Source				
	Q1	Q2	Q3	Q4	Q5
Men (*n*)	(1378)	(1378)	(1379)	(1378)	(1378)
Total fiber, g					
Median ± SE	11.7 ± 0.1	18.5 ± 0.1	24.3 ± 0.1	31.4 ± 0.1	43.9 ± 0.4
Interquartile range (Q1–Q3)	9.2–13.6	16.9–19.9	22.9–25.9	29.3–33.7	39.7–52.4
Carbohydrate: Total fiber					
Median ± SE	8.4 ± 0.04	11.1 ± 0.02	13.7 ± 0.02	16.7 ± 0.04	23.1 ± 0.3
Interquartile range (Q1–Q3)	7.3–9.2	10.5–11.8	13.1–14.3	15.8–17.7	20.7–27.3
Cereal fiber, g					
Median ± SE	1.6 ± 0.02	3.0 ± 0.01	4.6 ± 0.02	6.7 ± 0.02	11.2 ± 0.2
Interquartile range (Q1–Q3)	1.2–2.0	2.7–3.3	4.2–5.0	6.0–7.4	9.5–14.3
Vegetable fiber, g					
Median ± SE	2.8 ± 0.04	5.4 ± 0.02	7.7 ± 0.02	10.7 ± 0.03	16.4 ± 0.2
Interquartile range (Q1–Q3)	1.8–3.5	4.8–6.0	7.1–8.4	9.8–11.7	14.3–20.2
Fruit fiber, g	(2273)	(1172)	(1723)	(1723)	
Median ± SE	0.0 ± 0.0	0.1 ± 0.01	2.0 ± 0.03	8.0 ± 0.2	
Interquartile range (Q1–Q3)	0.0–0.0	0.01–0.2	1.2–3.0	5.5–12.1	
Women (*n*)	(1980)	(1980)	(1981)	(1980)	(1980)
Total fiber, g					
Median ± SE	9.7 ± 0.1	15.5 ± 0.04	20.5 ± 0.04	27.0 ± 0.1	39.2 ± 0.3
Interquartile range (Q1–Q3)	7.4–11.3	14.2–16.7	19.2–21.9	25.2–29.1	34.7–47.4
Carbohydrate: Total fiber					
Median ± SE	7.7 ± 0.03	10.3 ± 0.02	12.7 ± 0.02	15.6 ± 0.03	21.6 ± 0.2
Interquartile range (Q1–Q3)	6.6–8.5	9.7–10.8	12.0–13.2	14.8–16.6	19.3–26.0
Cereal fiber, g					
Median ± SE	1.3 ± 0.01	2.5 ± 0.01	3.8 ± 0.01	5.5 ± 0.02	9.6 ± 0.2
Interquartile range (Q1–Q3)	0.9–1.6	2.2–2.8	3.4–4.2	5.0–6.2	8.1–12.7
Vegetable fiber, g					
Median ± SE	1.8 ± 0.02	3.9 ± 0.01	5.8 ± 0.01	8.3 ± 0.02	13.5 ± 0.1
Interquartile range (Q1–Q3)	1.1–2.5	3.4–4.4	5.3–6.3	7.5–9.1	11.5–16.7
Fruit fiber, g	(2475)	(2474)	(2477)	(2475)	
Median ± SE	0.0 ± 0.0	0.5 ± 0.01	3.2 ± 0.02	8.9 ± 0.2	
Interquartile range (Q1–Q3)	0.0–0.0	0.2–1.1	2.4–4.1	7.0–13.7	

SE, standard error. ^1^ All of the analyses accounted for the effects of the complex sampling design and appropriate sampling weights of the national survey using the PROC SURVEY procedures in the SAS software.

**Table 3:** Associations of dietary fiber and its source with cardiovascular risk factors among Korean men ^1,2^.

	Quintile (Quartile) of Dietary Fiber and Its Source					*p* Trend
	Q1	Q2	Q3	Q4	Q5	
	OR	OR (95% CI)	OR (95% CI)	OR (95% CI)	OR (95% CI)	
**Total fiber**						
Obesity ^3^	1.00 (Ref)	0.84 (0.70,1.00)	0.96 (0.80,1.16)	0.97 (0.80,1.17)	1.16 (0.93,1.44)	0.041
Abdominal obesity ^3^	1.00 (Ref)	0.86 (0.71,1.06)	0.84 (0.69,1.03)	0.89 (0.73,1.10)	1.14 (0.90,1.44)	0.111
Metabolic syndrome	1.00 (Ref)	1.00 (0.79,1.28)	0.76 (0.60,0.98)	0.81 (0.62,1.06)	0.69 (0.53,0.92)	0.004
Hypercholesterolemia	1.00 (Ref)	0.98 (0.71,1.35)	1.01 (0.74,1.39)	1.25 (0.91,1.73)	1.08 (0.76,1.52)	0.393
Hypertension	1.00 (Ref)	1.25 (0.97,1.61)	1.00 (0.76,1.31)	1.12 (0.85,1.47)	0.88 (0.65,1.17)	0.128
Type 2 diabetes	1.00 (Ref)	0.95 (0.57,1.59)	1.00 (0.62,1.61)	0.95 (0.57,1.58)	0.70 (0.41,1.19)	0.148
**Carbohydrate: Total fiber**						
Obesity ^3^	1.00 (Ref)	0.88 (0.74,1.05)	0.84 (0.70,1.00)	0.67 (0.56,0.80)	0.76 (0.63,0.92)	0.002
Abdominal obesity ^3^	1.00 (Ref)	0.85 (0.70,1.03)	0.86 (0.72,1.04)	0.72 (0.59,0.88)	0.74 (0.61,0.91)	0.004
Metabolic syndrome	1.00 (Ref)	1.00 (0.80,1.26)	1.06 (0.85,1.32)	1.17 (0.92,1.50)	1.12 (0.88,1.43)	0.208
Hypercholesterolemia	1.00 (Ref)	0.98 (0.75,1.29)	0.82 (0.62,1.08)	0.77 (0.56,1.04)	0.89 (0.66,1.20)	0.309
Hypertension	1.00 (Ref)	1.02 (0.81,1.29)	1.07 (0.84,1.37)	1.14 (0.90,1.46)	0.88 (0.68,1.13)	0.399
Type 2 diabetes	1.00 (Ref)	0.88 (0.57,1.37)	0.97 (0.62,1.51)	0.92 (0.58,1.47)	1.14 (0.73,1.79)	0.481
**Cereal fiber**						
Obesity ^3^	1.00 (Ref)	1.00 (0.84,1.19)	0.92 (0.77,1.10)	0.95 (0.79,1.13)	0.91 (0.76,1.09)	0.254
Abdominal obesity ^3^	1.00 (Ref)	1.02 (0.84,1.23)	0.97 (0.80,1.18)	0.90 (0.74,1.10)	0.93 (0.77,1.14)	0.307
Metabolic syndrome	1.00 (Ref)	1.08 (0.86,1.37)	0.88 (0.69,1.13)	0.92 (0.72,1.18)	0.92 (0.72,1.19)	0.327
Hypercholesterolemia	1.00 (Ref)	0.97 (0.72,1.31)	0.94 (0.69,1.28)	1.20 (0.89,1.62)	1.07 (0.77,1.47)	0.392
Hypertension	1.00 (Ref)	0.99 (0.78,1.27)	0.99 (0.78,1.27)	0.96 (0.75,1.24)	1.01 (0.78,1.31)	0.963
Type 2 diabetes	1.00 (Ref)	0.91 (0.57,1.46)	1.09 (0.67,1.78)	1.03 (0.63,1.69)	0.92 (0.56,1.51)	0.823
**Vegetable fiber**						
Obesity ^3^	1.00 (Ref)	0.83 (0.70,1.00)	0.91 (0.76,1.09)	1.00 (0.83,1.19)	1.28 (1.05,1.55)	0.001
Abdominal obesity ^3^	1.00 (Ref)	0.77 (0.64,0.94)	0.96 (0.78,1.17)	0.92 (0.75,1.12)	1.04 (0.85,1.29)	0.229
Metabolic syndrome	1.00 (Ref)	0.91 (0.71,1.16)	0.97 (0.76,1.23)	0.85 (0.65,1.10)	0.96 (0.75,1.23)	0.791
Hypercholesterolemia	1.00 (Ref)	1.07 (0.79,1.44)	0.94 (0.70,1.25)	0.88 (0.65,1.20)	0.97 (0.71,1.34)	0.625
Hypertension	1.00 (Ref)	1.11 (0.85,1.45)	1.15 (0.89,1.49)	1.11 (0.85,1.43)	1.15 (0.87,1.52)	0.430
Type 2 diabetes	1.00 (Ref)	0.91 (0.54,1.52)	0.95 (0.58,1.54)	0.87 (0.52,1.47)	1.02 (0.62,1.68)	0.871
**Fruit fiber**						
Obesity ^3^	1.00 (Ref)	0.99 (0.84,1.17)	0.84 (0.72,0.98)	0.87 (0.74,1.02)		0.093
Abdominal obesity ^3^	1.00 (Ref)	0.99 (0.82,1.18)	0.78 (0.66,0.92)	0.74 (0.62,0.88)		0.001
Metabolic syndrome	1.00 (Ref)	0.97 (0.79,1.21)	0.78 (0.63,0.95)	0.76 (0.61,0.93)		0.011
Hypercholesterolemia	1.00 (Ref)	1.18 (0.89,1.58)	1.14 (0.88,1.49)	1.37 (1.04,1.79)		0.042
Hypertension	1.00 (Ref)	0.88 (0.69,1.12)	0.95 (0.76,1.18)	0.77 (0.61,0.97)		0.035
Type 2 diabetes	1.00 (Ref)	1.17 (0.78,1.77)	0.70 (0.45,1.07)	1.15 (0.79,1.68)		0.467

^1^ All of the analyses accounted for the effects of the complex sampling design and appropriate sampling weights of the national survey using the PROC SURVEY procedures in the SAS software. ^2^ Multiple logistic regression analyses were performed to estimate odds ratios (ORs), 95% confidence intervals (CIs), and *p* trend values for cardiovascular risk factors by quintile (or quartile) of each dietary measure, taking the lowest quintile (quartile) group as the reference (Ref) group. To calculate the *p* trend values across quintiles (or quartiles) of dietary measures, the median intake of a dietary measure in each quintile (or quartile) was entered as a continuous variable in the logistic regression model. Age (continuous), living area (urban or rural), education (elementary school, middle school, high school, or college or more), household income (lowest, medium-low, medium-high, or highest), current smoking (yes or no), current alcohol drinking (yes or no), physical activity (yes or no), BMI (continuous), and total energy intake (continuous) were controlled for in all of the models. ^3^ Adjusted for age, living area, education, household income, current smoking, current alcohol drinking, physical activity, and total energy intake.

**Table 4:** Associations of dietary fiber and its source with cardiovascular risk factors among Korean women ^1,2^.

	Quintile (Quartile) of Dietary Fiber and Its Source					*p* Trend
	Q1	Q2	Q3	Q4	Q5	
	OR	OR (95% CI)	OR (95% CI)	OR (95% CI)	OR (95% CI)	
**Total fiber**						
Obesity ^3^	1.00 (Ref)	1.04 (0.87,1.24)	0.98 (0.81,1.18)	1.10 (0.90,1.34)	1.05 (0.83,1.33)	0.596
Abdominal obesity ^3^	1.00 (Ref)	0.97 (0.79,1.20)	1.07 (0.87,1.31)	0.93 (0.74,1.17)	0.90 (0.70,1.17)	0.351
Metabolic syndrome	1.00 (Ref)	1.20 (0.91,1.57)	1.31 (1.01,1.70)	0.86 (0.63,1.16)	1.05 (0.76,1.46)	0.497
Hypercholesterolemia	1.00 (Ref)	0.98 (0.74,1.28)	1.20 (0.91,1.59)	0.98 (0.74,1.30)	1.17 (0.84,1.62)	0.412
Hypertension	1.00 (Ref)	1.04 (0.77,1.42)	1.21 (0.91,1.62)	0.90 (0.65,1.25)	0.96 (0.67,1.38)	0.506
Type 2 diabetes	1.00 (Ref)	0.68 (0.37,1.25)	0.82 (0.45,1.51)	0.79 (0.39,1.59)	0.92 (0.48,1.76)	0.918
**Carbohydrate: Total fiber**						
Obesity ^3^	1.00 (Ref)	0.90 (0.75,1.07)	0.93 (0.78,1.11)	0.95 (0.80,1.13)	0.91 (0.76,1.10)	0.541
Abdominal obesity ^3^	1.00 (Ref)	0.89 (0.73,1.09)	0.98 (0.80,1.19)	1.04 (0.85,1.27)	1.01 (0.83,1.24)	0.490
Metabolic syndrome	1.00 (Ref)	0.95 (0.73,1.24)	1.18 (0.91,1.53)	1.13 (0.88,1.45)	1.24 (0.94,1.63)	0.066
Hypercholesterolemia	1.00 (Ref)	0.92 (0.73,1.17)	0.97 (0.75,1.25)	0.88 (0.68,1.14)	0.88 (0.67,1.15)	0.333
Hypertension	1.00 (Ref)	1.14 (0.86,1.51)	1.35 (1.04,1.76)	1.26 (0.94,1.71)	1.07 (0.78,1.45)	0.678
Type 2 diabetes	1.00 (Ref)	1.05 (0.59,1.88)	0.63 (0.35,1.13)	1.00 (0.56,1.77)	0.84 (0.45,1.57)	0.581
**Cereal fiber**						
Obesity ^3^	1.00 (Ref)	0.98 (0.82,1.17)	0.92 (0.78,1.10)	0.96 (0.80,1.14)	1.05 (0.87,1.28)	0.476
Abdominal obesity ^3^	1.00 (Ref)	0.94 (0.77,1.14)	0.86 (0.70,1.05)	0.99 (0.81,1.22)	1.06 (0.84,1.32)	0.311
Metabolic syndrome	1.00 (Ref)	1.14 (0.88,1.49)	1.19 (0.92,1.53)	1.13 (0.86,1.49)	1.08 (0.81,1.42)	0.923
Hypercholesterolemia	1.00 (Ref)	0.92 (0.71,1.19)	0.84 (0.64,1.09)	1.00 (0.78,1.28)	0.85 (0.64,1.11)	0.419
Hypertension	1.00 (Ref)	1.07 (0.79,1.44)	1.33 (0.98,1.80)	1.28 (0.95,1.72)	1.24 (0.91,1.69)	0.174
Type 2 diabetes	1.00 (Ref)	0.71 (0.41,1.21)	0.67 (0.34,1.30)	0.65 (0.37,1.14)	0.62 (0.34,1.11)	0.188
**Vegetable fiber**						
Obesity ^3^	1.00 (Ref)	1.01 (0.85,1.21)	1.01 (0.84,1.21)	0.96 (0.80,1.15)	1.18 (0.98,1.44)	0.092
Abdominal obesity ^3^	1.00 (Ref)	1.08 (0.88,1.31)	0.98 (0.80,1.20)	0.94 (0.76,1.17)	1.03 (0.83,1.28)	0.934
Metabolic syndrome	1.00 (Ref)	1.03 (0.78,1.37)	1.05 (0.80,1.38)	1.10 (0.84,1.45)	1.05 (0.79,1.39)	0.728
Hypercholesterolemia	1.00 (Ref)	0.82 (0.62,1.08)	0.85 (0.65,1.12)	1.05 (0.80,1.37)	0.85 (0.64,1.14)	0.731
Hypertension	1.00 (Ref)	1.08 (0.79,1.47)	1.31 (0.96,1.77)	1.22 (0.90,1.64)	1.13 (0.83,1.53)	0.555
Type 2 diabetes	1.00 (Ref)	0.69 (0.35,1.37)	0.61 (0.32,1.14)	1.15 (0.64,2.05)	1.48 (0.84,2.59)	0.017
**Fruit fiber**						
Obesity ^3^	1.00 (Ref)	0.99 (0.85,1.15)	0.80 (0.69,0.93)	0.85 (0.73,1.00)		0.029
Abdominal obesity ^3^	1.00 (Ref)	0.97 (0.82,1.16)	0.82 (0.69,0.98)	0.82 (0.67,0.99)		0.026
Metabolic syndrome	1.00 (Ref)	0.95 (0.76,1.18)	1.09 (0.87,1.36)	0.88 (0.69,1.13)		0.321
Hypercholesterolemia	1.00 (Ref)	1.06 (0.83,1.35)	1.17 (0.93,1.47)	1.18 (0.93,1.49)		0.210
Hypertension	1.00 (Ref)	0.71 (0.55,0.93)	0.92 (0.72,1.18)	0.70 (0.54,0.90)		0.052
Type 2 diabetes	1.00 (Ref)	0.78 (0.47,1.30)	0.93 (0.56,1.54)	0.51 (0.28,0.91)		0.030

^1^ All of the analyses accounted for the effects of the complex sampling design and appropriate sampling weights of the national survey using the PROC SURVEY procedures in the SAS software. ^2^ Multiple logistic regression analyses were performed to estimate odds ratios (ORs), 95% confidence intervals (CIs), and *p* trend values for cardiovascular risk factors by quintile (or quartile) of each dietary measure, taking the lowest quintile (quartile) group as the reference (Ref) group. To calculate the *p* trend values across quintiles (or quartiles) of dietary measures, the median intake of a dietary measure in each quintile (or quartile) was entered as a continuous variable in the logistic regression model. Age (continuous), living area (urban or rural), education (elementary school, middle school, high school, or college or more), household income (lowest, medium-low, medium-high, or highest), current smoking (yes or no), current alcohol drinking (yes or no), physical activity (yes or no), BMI (continuous), and total energy intake (continuous) were controlled for in all of the models. ^3^ Adjusted for age, living area, education, household income, current smoking, current alcohol drinking, physical activity, and total energy intake.

**Table 5:** Associations of total fruit and whole fruit consumption with cardiovascular risk factors among Korean adults by sex ^1,2^.

	Quartile of Total Fruit and Whole Fruit Consumption				
	Q1	Q2	Q3	Q4	
	OR	OR (95% CI)	OR (95% CI)	OR (95% CI)	*p* Trend
**Men**					
**Total fruit**, g	(*n* = 2236)	(*n* = 1209)	(*n* = 1723)	(*n* = 1723)	
Mean ± SE (CV)	0.0	12.5 ± 15.1 (121.0)	155.3 ± 58.4 (37.6)	563.2 ± 323.1 (57.4)	
Median (Interquartile range, Q1–Q3)	0.0 (0.0–0.0)	5.7 (1.1–18.3)	151.4 (105.7–207.5)	462.1 (339.2–672.1)	
Obesity ^3^	1.00 (Ref)	1.02 (0.86,1.20)	0.86 (0.74,1.00)	0.84 (0.72,0.98)	0.013
Abdominal obesity ^3^	1.00 (Ref)	0.94 (0.78,1.12)	0.80 (0.68,0.94)	0.74 (0.63,0.88)	0.001
Metabolic syndrome	1.00 (Ref)	0.97 (0.79,1.20)	0.82 (0.66,1.00)	0.73 (0.59,0.89)	0.001
Hypercholesterolemia	1.00 (Ref)	1.18 (0.88,1.57)	1.15 (0.88,1.50)	1.33 (1.02,1.73)	0.066
Hypertension	1.00 (Ref)	0.91 (0.72,1.16)	0.88 (0.70,1.09)	0.78 (0.62,0.98)	0.041
Type 2 diabetes	1.00 (Ref)	1.18 (0.78,1.78)	0.69 (0.45,1.06)	1.09 (0.75,1.60)	0.823
**Whole fruit**, g	(*n* = 2617)	(*n* = 828)	(*n* = 1723)	(*n* = 1723)	
Mean ± SE (CV)	0.0	8.1 ± 8.4 (103.8)	135.1 ± 58.5 (43.3)	537.5 ± 323.6 (60.2)	
Median (Interquartile range, Q1–Q3)	0.0 (0.0–0.0)	5.0 (1.8–11.5)	136.5 (82.3–186.3)	435.2 (305.2–642.0)	
Obesity ^3^	1.00 (Ref)	1.01 (0.85,1.22)	0.92 (0.80,1.06)	0.85 (0.73,0.99)	0.023
Abdominal obesity ^3^	1.00 (Ref)	0.99 (0.81,1.21)	0.81 (0.69,0.95)	0.78 (0.66,0.92)	0.002
Metabolic syndrome	1.00 (Ref)	0.93 (0.74,1.18)	0.84 (0.68,1.03)	0.68 (0.55,0.83)	<0.001
Hypercholesterolemia	1.00 (Ref)	1.20 (0.88,1.63)	1.05 (0.82,1.35)	1.29 (0.99,1.67)	0.098
Hypertension	1.00 (Ref)	0.96 (0.73,1.26)	0.88 (0.71,1.09)	0.84 (0.68,1.05)	0.130
Type 2 diabetes	1.00 (Ref)	1.07 (0.65,1.77)	0.71 (0.47,1.09)	0.88 (0.60,1.28)	0.467
**Women**					
**Total fruit**, g	(*n* = 2475)	(*n* = 2475)	(*n* = 2476)	(*n* = 2475)	
Mean ± SE (CV)	0.03 ± 0.12 (365.7)	58.2 ± 41.9 (71.9)	216.5 ± 54.3 (25.1)	615.1 ± 348.1 (56.6)	
Median (Interquartile range, Q1–Q3)	0.0 (0.0–0.0)	59.0 (13.2–94.5)	209.6 (167.2–262.2)	509.3 (394.3–706.3)	
Obesity ^3^	1.00 (Ref)	0.95 (0.82,1.10)	0.84 (0.72,0.97)	0.86 (0.73,1.01)	0.062
Abdominal obesity ^3^	1.00 (Ref)	0.97 (0.82,1.14)	0.77 (0.64,0.92)	0.86 (0.70,1.04)	0.084
Metabolic syndrome	1.00 (Ref)	1.12 (0.89,1.40)	0.98 (0.79,1.22)	0.89 (0.69,1.15)	0.146
Hypercholesterolemia	1.00 (Ref)	1.10 (0.87,1.40)	1.00 (0.79,1.26)	1.18 (0.92,1.51)	0.271
Hypertension	1.00 (Ref)	0.74 (0.57,0.96)	0.93 (0.72,1.20)	0.68 (0.53,0.88)	0.023
Type 2 diabetes	1.00 (Ref)	0.89 (0.54,1.47)	0.75 (0.43,1.28)	0.72 (0.41,1.26)	0.255
**Whole fruit**, g	(*n* = 2663)	(*n* = 2287)	(*n* = 2476)	(*n* = 2475)	
Mean ± SE (CV)	0.0	47.3 ± 37.3 (78.8)	197.7 ± 53.8 (27.2)	588.4 ± 345.1 (58.7)	
Median (Interquartile range, Q1–Q3)	0.0 (0.0–0.0)	44.6 (6.2–78.0)	192.2 (151.4–236.8)	482.9 (375.9–666.6)	
Obesity ^3^	1.00 (Ref)	0.92 (0.79,1.07)	0.88 (0.75,1.02)	0.86 (0.73,1.00)	0.092
Abdominal obesity ^3^	1.00 (Ref)	0.89 (0.75,1.06)	0.80 (0.67,0.96)	0.84 (0.69,1.02)	0.120
Metabolic syndrome	1.00 (Ref)	0.96 (0.77,1.20)	0.97 (0.78,1.20)	0.83 (0.65,1.06)	0.126
Hypercholesterolemia	1.00 (Ref)	1.20 (0.95,1.51)	0.91 (0.72,1.14)	1.23 (0.97,1.56)	0.227
Hypertension	1.00 (Ref)	0.72 (0.55,0.94)	0.96 (0.76,1.22)	0.69 (0.54,0.89)	0.033
Type 2 diabetes	1.00 (Ref)	0.96 (0.58,1.60)	0.70 (0.41,1.18)	0.80 (0.46,1.41)	0.421

CV, coefficient of variation; SE, standard error. ^1^ All of the analyses accounted for the effects of the complex sampling design and appropriate sampling weights of the national survey using the PROC SURVEY procedures in the SAS software. ^2^ Multiple logistic regression analyses were performed to estimate odds ratios (ORs), 95% confidence intervals (CIs), and *p* trend values for cardiovascular risk factors by quartile of total fruit and whole fruit consumption, taking the lowest quartile group as the reference (Ref) group. To calculate the *p* trend values across quartiles of total fruit and whole fruit consumption, the median intake in each quartile was entered as a continuous variable in the logistic regression model. Age (continuous), living area (urban or rural), education (elementary school, middle school, high school, or college or more), household income (lowest, medium-low, medium-high, or highest), current smoking (yes or no), current alcohol drinking (yes or no), physical activity (yes or no), BMI (continuous), and total energy intake (continuous) were controlled for in all of the models. ^3^ Adjusted for age, living area, education, household income, current smoking, current alcohol drinking, physical activity, and total energy intake.

## Data Availability

Publicly available datasets were analyzed in this study. This data can be found here: https://knhanes.cdc.go.kr.

## References

[1] Ludwig D.S., Hu F.B., Tappy L., Brand-Miller J. (2018). Dietary carbohydrates: Role of quality and quantity in chronic disease. BMJ.

[2] Aleixandre A., Miguel M. (2008). Dietary fiber in the prevention and treatment of metabolic syndrome: A review. Crit. Rev. Food Sci. Nutr..

[3] Aune D., Giovannucci E., Boffetta P., Fadnes L.T., Keum N., Norat T., Greenwood D.C., Riboli E., Vatten L.J., Tonstad S. (2017). Fruit and vegetable intake and the risk of cardiovascular disease, total cancer and all-cause mortality-a systematic review and dose-response meta-analysis of prospective studies. Int. J. Epidemiol..

[4] Hu E.A., Pan A., Malik V., Sun Q. (2012). White rice consumption and risk of type 2 diabetes: Meta-analysis and systematic review. BMJ.

[5] Xi B., Huang Y., Reilly K.H., Li S., Zheng R., Barrio-Lopez M.T., Martinez-Gonzalez M.A., Zhou D. (2015). Sugar-sweetened beverages and risk of hypertension and CVD: A dose-response meta-analysis. Br. J. Nutr..

[6] Reynolds A., Mann J., Cummings J., Winter N., Mete E., Te Morenga L. (2019). Carbohydrate quality and human health: A series of systematic reviews and meta-analyses. Lancet.

[7] Aune D., Keum N., Giovannucci E., Fadnes L.T., Boffetta P., Greenwood D.C., Tonstad S., Vatten L.J., Riboli E., Norat T. (2016). Whole grain consumption and risk of cardiovascular disease, cancer, and all cause and cause specific mortality: Systematic review and dose-response meta-analysis of prospective studies. BMJ.

[8] Korczak R., Marquart L., Slavin J.L., Ringling K., Chu Y., O'Shea M., Harriman C., Toups K., de Vries J., Jacques P. (2016). Thinking critically about whole-grain definitions: Summary report of an interdisciplinary roundtable discussion at the 2015 Whole Grains Summit. Am. J. Clin. Nutr..

[9] Dominianni C., Sinha R., Goedert J.J., Pei Z., Yang L., Hayes R.B., Ahn J. (2015). Sex, body mass index, and dietary fiber intake influence the human gut microbiome. PLoS ONE.

[10] Tilg H., Kaser A. (2011). Gut microbiome, obesity, and metabolic dysfunction. J. Clin. Invest..

[11] Zhou Q., Wu J., Tang J., Wang J.J., Lu C.H., Wang P.X. (2015). Beneficial effect of higher dietary fiber intake on plasma HDL-C and TC/HDL-C ratio among Chinese rural-to-urban migrant workers. Int. J. Environ. Res. Public Health.

[12] Aljuraiban G.S., Griep L.M., Chan Q., Daviglus M.L., Stamler J., Van Horn L., Elliott P., Frost G.S. (2015). Total, insoluble and soluble dietary fibre intake in relation to blood pressure: The Intermap Study. Br. J. Nutr..

[13] Moreno Franco B., Leon Latre M., Andrés Esteban E.M., Ordovás J.M., Casasnovas J.A., Peñalvo J.L. (2014). Soluble and insoluble dietary fibre intake and risk factors for metabolic syndrome and cardiovascular disease in middle-aged adults: The AWHS cohort. Nutr. Hosp..

[14] Chen J.P., Chen G.C., Wang X.P., Qin L., Bai Y. (2018). Dietary fiber and metabolic syndrome: A meta-analysis and review of related mechanisms. Nutrients.

[15] Lloyd-Jones D.M., Hong Y., Labarthe D., Mozaffarian D., Appel L.J., Van Horn L., Greenlund K., Daniels S., Nichol G., Tomaselli G.F. (2010). Defining and setting national goals for cardiovascular health promotion and disease reduction: The American Heart Association’s strategic Impact Goal through 2020 and beyond. Circulation.

[16] Fontanelli M.M., Micha R., Sales C.H., Liu J., Mozaffarian D., Fisberg R.M. (2019). Application of the </=10:1 carbohydrate-to-fiber ratio to identify healthy grain foods and its association with cardiometabolic risk factors.. Eur. J. Nutr..

[17] AlEssa H.B., Cohen R., Malik V.S., Adebamowo S.N., Rimm E.B., Manson J.E., Willett W.C., Hu F.B. (2018). Carbohydrate quality and quantity and risk of coronary heart disease among US women and men. Am. J. Clin. Nutr..

[18] Hosseinpour-Niazi S., Mirmiran P., Mirzaei S., Azizi F. (2015). Cereal, fruit and vegetable fibre intake and the risk of the metabolic syndrome: A prospective study in the Tehran Lipid and Glucose Study. J. Hum. Nutr. Diet..

[19] Eshak E.S., Iso H., Date C., Kikuchi S., Watanabe Y., Wada Y., Wakai K., Tamakoshi A., JACC Study Group (2010). Dietary fiber intake is associated with reduced risk of mortality from cardiovascular disease among Japanese men and women. J. Nutr..

[20] Song S., Lee J.E., Song W.O., Paik H.Y., Song Y. (2014). Carbohydrate intake and refined-grain consumption are associated with metabolic syndrome in the Korean adult population. J. Acad. Nutr. Diet..

[21] Kweon S., Kim Y., Jang M.J., Kim Y., Kim K., Choi S., Chun C., Khang Y.H., Oh K. (2014). Data resource profile: The Korea National Health and Nutrition Examination Survey (KNHANES). Int. J. Epidemiol..

[22] National Institute of Agricultural Sciences (2011). Food Composition Table.

[23] National Institute of Agricultural Sciences (2016). Food Composition Table.

[24] International Obesity Taskforce (2000). The Asia-Pacific Perspective: Redefining Obesity and Its Treatment.

[25] Lee S.Y., Park H.S., Kim D.J., Han J.H., Kim S.M., Cho G.J., Kim D.Y., Kwon H.S., Kim S.R., Lee C.B. (2007). Appropriate waist circumference cutoff points for central obesity in Korean adults. Diabetes Res. Clin. Pract..

[26] National Cholesterol Education Program (NCEP) Expert Panel on Detection, Evaluation, and Treatment of High Blood Cholesterol in Adults (Adult Treatment Panel III) (2002). Third report of the National Cholesterol Education Program (NCEP) Expert Panel on Detection, Evaluation, and Treatment of High Blood Cholesterol in Adults (Adult Treatment Panel III) final report. Circulation.

[27] Kim H.C., Ihm S.H., Kim G.H., Kim J.H., Kim K.I., Lee H.Y., Lee J.H., Park J.M., Park S., Pyun W.B. (2019). 2018 Korean Society of Hypertension guidelines for the management of hypertension: Part I-epidemiology of hypertension. Clin. Hypertens..

[28] Korean Diabetes Association (2019). 2019 Treatment Guideline for Diabetes.

[29] Morimoto N., Kasuga C., Tanaka A., Kamachi K., Ai M., Urayama K.Y., Tanaka A. (2018). Association between dietary fibre: Carbohydrate intake ratio and insulin resistance in Japanese adults without type 2 diabetes. Br. J. Nutr..

[30] McKeown N.M., Meigs J.B., Liu S., Saltzman E., Wilson P.W.F., Jacques P.F. (2004). Carbohydrate nutrition, insulin resistance, and the prevalence of the metabolic syndrome in the Framingham Offspring Cohort. Diabetes Care.

[31] Du H., van der A.D., Boshuizen H.C., Forouhi N.G., Wareham N.J., Halkjaer J., Tjønneland A., Overvad K., Jakobsen M.U., Boeing H. (2010). Dietary fiber and subsequent changes in body weight and waist circumference in European men and women. Am. J. Clin. Nutr..

[32] Liu S., Willett W.C., Manson J.E., Hu F.B., Rosner B., Colditz G. (2003). Relation between changes in intakes of dietary fiber and grain products and changes in weight and development of obesity among middle-aged women. Am. J. Clin. Nutr..

[33] Kim Y., Je Y. (2016). Dietary fibre intake and mortality from cardiovascular disease and all cancers: A meta-analysis of prospective cohort studies. Arch. Cardiovasc. Dis..

[34] Anderson J.W., Baird P., Davis R.H., Ferreri S., Knudtson M., Koraym A., Waters V., Williams C.L. (2009). Health benefits of dietary fiber. Nutr. Rev..

[35] The Korean Nutrition Society (2015). Dietary Reference Intakes for Koreans 2015.

[36] Nakaji S., Sugawara K., Saito D., Yoshioka Y., MacAuley D., Bradley T., Kernohan G., Baxter D. (2002). Trends in dietary fiber intake in Japan over the last century. Eur. J. Nutr..

[37] Wang H.J., Wang Z.H., Zhang J.G., Du W.W., Su C., Zhang Z., Zhai F.Y., Zhang B. (2014). Trends in dietary fiber intake in Chinese aged 45 years and above, 1991–2011. Eur. J. Clin. Nutr..

[38] Hosseinpour-Niazi S., Mirmiran P., Sohrab G., Hosseini-Esfahani F., Azizi F. (2011). Inverse association between fruit, legume, and cereal fiber and the risk of metabolic syndrome: Tehran Lipid and Glucose Study. Diabetes Res. Clin. Pract..

[39] Hong S., Song Y., Lee K.H., Lee H.S., Lee M., Jee S.H., Joung H. (2012). A fruit and dairy dietary pattern is associated with a reduced risk of metabolic syndrome. Metabolism.

[40] Kim J., Kim J. (2018). Association between fruit and vegetable consumption and risk of hypertension in middle-aged and older Korean adults. J. Acad. Nutr. Diet..

[41] Choi A., Ha K., Joung H., Song Y. (2019). Frequency of consumption of whole fruit, not fruit juice, is associated with reduced prevalence of obesity in Korean adults. J. Acad. Nutr. Diet..

[42] Shin J.H., Jung S., Kim S.A., Kang M.S., Kim M.S., Joung H., Hwang G.S., Shin D.M. (2019). Differential effects of typical Korean versus American-style diets on gut microbial composition and metabolic profile in healthy overweight Koreans: A randomized crossover trial. Nutrients.

[43] Schroeder N., Park Y.H., Kang M.S., Kim Y., Ha G.K., Kim H.R., Yates A.A., Caballero B. (2015). A randomized trial on the effects of 2010 Dietary Guidelines for Americans and Korean diet patterns on cardiovascular risk factors in overweight and obese adults. J. Acad. Nutr. Diet..

[44] Dinu M., Pagliai G., Angelino D., Rosi A., Dall’Asta M., Bresciani L., Ferraris C., Guglielmetti M., Godos J., Del Bo’ C. (2020). Effects of popular diets on anthropometric and cardiometabolic parameters: An umbrella review of meta-analyses of randomized controlled trials. Adv. Nutr..

[45] Squadrito F., Marini H., Bitto A., Altavilla D., Polito F., Adamo E.B., D'Anna R., Arcoraci V., Burnett B.P., Minutoli L. (2013). Genistein in the metabolic syndrome: Results of a randomized clinical trial. J. Clin. Endocrinol. Metab..

[46] Rees A., Dodd G.F., Spencer J.P.E. (2018). The effects of flavonoids on cardiovascular health: A review of human intervention trials and implications for cerebrovascular function. Nutrients.

[47] Ma L., Liu G., Ding M., Zong G., Hu F.B., Willett W.C., Rimm E.B., Manson J.E., Sun Q. (2020). Isoflavone intake and the risk of coronary heart disease in US men and women: Results from 3 prospective cohort studies. Circulation.

